# A Practical Treatise on the Physical Exploration of the Chest, and the Diagnosis of the Diseases of the Respiratory Organs

**Published:** 1866-11

**Authors:** 


A Practical Treatise on the Physical Exploration of the Chest, and the Diagnosis of the Diseases of the Respiratory Organs. By Austin Flint, M.D., Prof. Prin. and Prac. of Med., in the Bellevue Hospital Med. College, and the Long Island College Hospital; Fellow of New York Medical Academy, etc. Second edition, revised. Philadelphia: Henry C. Lea. 1866.

This is a well-executed volume, of about 600 pages. The first edition has been long enough before the profession to have its merits well known. The present edition exhibits indications of having been carefully revised and, in some instances, altered in accordance with the more extended opportunities for observation which the author has enjoyed for the last few years in the Bellevue and Long Island Hospitals. The contents of the book are sufficiently indicated by its title; and we freely commend it to the study of the profession.

For sale by W. B. Keen & Co., 148 Lake Street.

